# Autophagy Modulators in Cancer: Focus on Cancer Treatment

**DOI:** 10.3390/life11080839

**Published:** 2021-08-17

**Authors:** Hye Jin Nam

**Affiliations:** Drug Discovery Platform Research Center, Therapeutics and Biotechnology Division, Korea Research Institute of Chemical Technology, Daejeon 34114, Korea; hjnam@krict.re.kr; Tel.: +82-42-860-7456

**Keywords:** autophagy, autophagy enhancer, autophagy inhibitor, cancer treatment

## Abstract

Uncontrolled autophagy has been associated with the development and progression of various cancers that are resistant to cancer therapy. Therefore, many efforts to modulate uncontrolled autophagy as a cancer treatment have been attempted, from basic science to clinical trials. However, it remains difficult to equally apply autophagy modulators to cancer therapy because autophagy is a double-edged sword in cancer: it can be tumor-suppressive or tumor-protective. Therefore, the precise mechanisms of autophagy modulators and their varied responsiveness to each cancer type should be addressed in detail. This study will describe the precise mechanisms of developing various autophagy modulators, their current therapeutic applications and future perspectives.

## 1. Introduction

Autophagy (self-eating) is defined as a homeostatic process that enables the lysosomal degradation of unnecessary or dysfunctional organelles or proteins [1]. There are three types of autophagy: microautophagy, chaperone-mediated autophagy (CMA) and macroautophagy [2]. Microautophagy is involved with the direct uptake of cargo by the lysosome, and little is known about its mechanism [3]. CMA is a selective process and involves substrates with specific motifs (KFERQ or similar KFERQ generated by modification). Heat-shock cognate chaperone of 70 kDa (Hsc70) recognizes the KFERQ motif of the substrate and binds and moves together to the lysosomal membrane. Chaperone selectively binds to LAMP2A on the lysosomal membrane and internalizes the substrate into the lysosome through LAMP2A. Thus, CMA has not only selective substrates but also specific machinery components [4].

**Figure 1:**
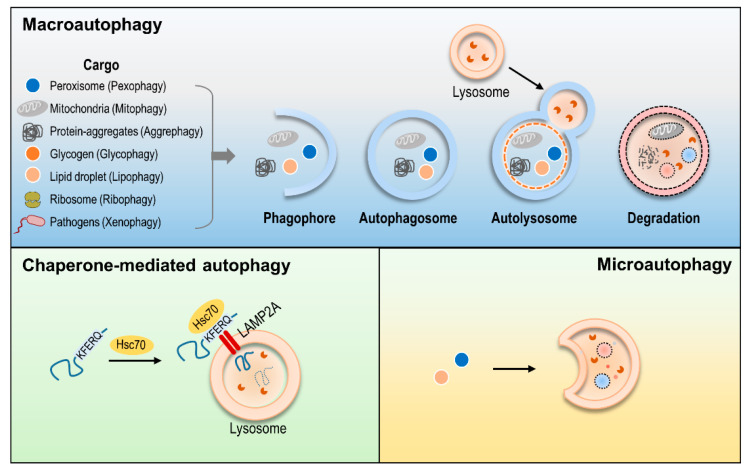
Schematic diagram of autophagic progression. There are three types of autophagy in mammalian cells: macroautophagy, microautophagy and chaperone-mediated autophagy. When macroautophagy is induced, cytoplasmic materials are engulfed by double membranes (phagophore), closed (autophagosome), fused with the lysosome (autolysosome) and degraded. The KFERQ motif is recognized by Hsc70 and binds with LAMP2A in CMA. LAMP2A mediates the translocation of the substrate into lysosomes. Microautophagy directly uptakes the cytoplasmic cargo.

Macroautophagy, usually considered as “autophagy” and thereafter referred to as autophagy, is primarily induced by nutrient starvation (e.g., glucose starvation or amino acid starvation) [5]. Cells deal with starvation by recycling cellular organelles and proteins and then generating energy. Autophagy is initiated with the formation of a phagophore, which is also known as an isolation membrane. The membrane source of phagophore has been proposed to originate from the smooth endoplasmic reticulum (ER), mitochondria or plasma membrane [6]. The conjugation of LC3 to phosphatidylethanolamine (PE) yields recruitment to the nascent phagophore structure [7]. The precursor proLC3 is cleaved by Atg4 protease into LC3-I with a C-terminal glycine residue, and the C-terminus is then conjugated to the polar head of PE by Atg complex to form LC3-II. The lipidated LC3 (LC3-II) is localized on the membrane’s inner and outer sides and contributes to the expansion and closure of the phagophore. As LC3-II migrates faster than LC3-I in gel electrophoresis experiments, it is widely used as a marker for assessing autophagy induction [5]. Phagophore grows, engulfs cytosolic components and closes to become an autophagosome. Then, the autophagosome fuses with a lysosome and degrades sequestered contents. Cellular response to nutrient deprivation is thought to be non-selective autophagy and usually involves random uptake of cytoplasm. Conversely, selective autophagy selectively eliminates specific cellular components, such as damaged organelles, or protein aggregates [8]. Moreover, pathogens are targeted for degradation by selective autophagy. Cargo-specific names have been used to classify the various types of selective autophagy (e.g., pexophagy, mitophagy, aggrephagy, glycophagy, lipophagy ribophagy and xenophagy) [9,10]. In particular, mitophagy is strongly associated with tumorigenesis [11,12] (Figure 1).

Autophagy-modulating drugs have been increasingly used in clinical trials; simultaneously, many phenotypic screens are being conducted for new drug discovery, which can modulate autophagy for therapeutic purposes. However, it is not easy to screen and identify new autophagy-modulating chemicals. The brief analysis of the role of autophagy modulators through LC3-II or autophagosome accumulation during drug screening may be misleading. An increase in LC3-II or autophagosomes can be interpreted as autophagy induction and blockade: an increase in the rate of autophagosome formation or a decrease in the rate of autophagosome clearance following fusion with lysosomes. To solve this problem, autophagic flux probes, such as GFP-LC3-RFP-LC3ΔG or mRFP-GFP-tagged LC3, were developed and used for high-throughput screening to identify autophagy modulators [13,14]. Autophagy flux was quantitatively monitored by calculating the GFP/RFP ratio or LC3 puncta: the decreased GFP/RFP ratio is detected by the autophagy inducer, whereas the autophagy inhibitor detects the increased GFP/RFP ratio. However, an assay using an autophagic flux probe has a limitation, as it does not distinguish whether the autophagy modulators inhibit the initiation step or lysosomal fusion step [15]. To apply autophagy modulators as therapeutics, more research beyond screening is required to determine which steps of autophagy are regulated by these chemicals. Through various autophagy screening methods, researchers are discovering novel autophagy modulators and/or adding new functions as autophagy modulators to existing drugs [15].

## 2. Autophagy: Tumor Suppressor or Promoter?

In normal cells, autophagy contributes to the maintenance of homeostasis. Autophagy is a mechanism to deal with starvation, but it also plays an essential role in removing substances that can be toxic to cells. For instance, autophagy is induced in response to reactive oxygen species (ROS) to remove them and protect the cells from apoptosis, whereas autophagy impairment causes the accumulation of oxidative stress [16,17]. Moreover, since ROS is involved in DNA damage and genetic instability, the removal of ROS by autophagy may be critical for blocking the transformation of normal cells [18,19].

However, in cancer cells, the role of autophagy has remained controversial. Autophagy has been reported to have both antitumor and tumor-promoting effects in cancers. Since the role of autophagy varies according to the cancer stage and tumor type, it is necessary first to check how autophagy is dysregulated in target cancers. Due to the opposite effects of autophagy, either inhibitors or inducers of autophagy could be exploited for cancer therapy depending on cancer context, respectively.

In the early stages of cancer development, autophagy is believed to play a protective role against cancer initiation. Inhibition of autophagy or defects of autophagy can lead to impaired removal of toxic materials such as damaged organelles, unfolded proteins or ROS. Here, inducing autophagy might serve as a cancer prevention or treatment. Additionally, *Beclin1*, which is the core subunit of the PI3K complex and is involved in initiation of autophagosome [20], heterozygote mice exhibited spontaneous development of malignant tumors, indicating that autophagy inhibition can induce tumorigenesis. Furthermore, decreased expression of autophagic genes (Atg5, Beclin1, Atg7) and autophagic activity was observed in hepatocellular carcinoma (HCC) [21]. In glioblastoma (GBM), low levels of ULK2 transcripts by DNA methylation were reported [22]. ULK2 overexpression induced autophagy and inhibited astrocyte transformation and tumor growth in glioblastoma. Altogether, these studies indicate that autophagy genes function as tumor suppressors in several tumors.

However, tumor-promoting effects of autophagy are more prominent in various cancers. Once the carcinogenic phenotype is established, cancer cells exploit autophagy mechanisms to satisfy their energy requirements. Particularly, autophagy contributes to the adaptation and survival of cancer cells in unfavorable conditions such as hypoxia or nutrient-deficient conditions [23]. Therefore, inducing autophagy in this situation may rather promote tumor progression. Notably, enhanced autophagy flux was observed in various tumors.

Additionally, autophagy has also been determined to promote the invasion of hepatocellular carcinoma cells through the activation of epithelial mesenchymal transition (EMT) [24]. Autophagy is also associated with the metastatic ability of pancreatic cancer [25]. These studies suggest that autophagy is critical for cancer metastasis [24]. However, it was reported that autophagy induction in glioblastoma cells rather impairs migration and invasion [26]. Interestingly, SNAIL, which is responsible for glioblastoma cell movement, was degraded upon autophagy induction. This is another example showing that autophagy does not have the same effect on all cancer types.

Autophagy is also related to cancer stem cells (CSCs) in various cancers such as breast, ovarian, liver and brain cancer. Basal levels of autophagy are required to maintain the balance between pluripotency and differentiation in CSCs. Intriguingly, changes in the basal levels of autophagy by either autophagy inducer or inhibitor can result in a decrease in pluripotency or an increase in differentiation and senescence in CSCs [27].

Several studies have already reported that autophagy flux is enhanced in various CSCs, and, in this case, suppressing autophagy has a positive effect in terms of cancer treatment. In mammosphere conditions, a culture system in which mammary CSCs/progenitor cells can be propagated, a greater autophagy flux was displayed than in normal adherent culture conditions [28]. The knockdown of Beclin1 impaired CSC maintenance and proliferations [28]. Additionally, ovarian CSCs have enhanced autophagic flux, and the inhibition of autophagy decreased the self-renewal ability and chemoresistance of ovarian CSCs [29]. It has been reported that the enhanced autophagy flux in liver CSCs contributes to the adaptation of liver CSCs to the tumor microenvironment, such as hypoxia and nutrient-deficient conditions [30]. Therefore, autophagy inhibitors may make liver CSCs difficult to survive in an unfavorable tumor microenvironment, which may help improve anticancer therapeutic effects.

CSC resistance to chemotherapy is also associated with autophagy [31]. Many studies have shown that combining cytotoxic drugs and autophagy inducers or inhibitors increases the sensitivity of CSCs. For instance, autophagy induction by rapamycin promoted cell differentiation and made GSCs sensitive to radiation therapy [32]. Azathioprine, an immunosuppressant for rheumatoid arthritis or Crohn’s disease, induces autophagy through mTORC1 [33] and also sensitizes GBM to chemotherapy or radiotherapy [34]. In terms of autophagy inhibition, chloroquine was able to increase the chemosensitivity of glioma cells to temozolomide [35]. Altogether, autophagy modulators can be used for cancer treatment because the imbalance in autophagy leads to cancer cell death.

## 3. Modulators of Initial Autophagy and Cancer Treatment

**Figure 2:**
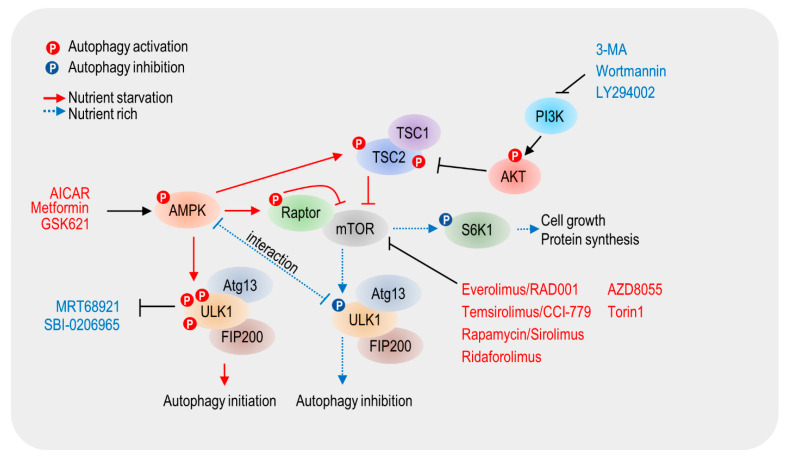
Signaling cascades and modulators that regulate autophagy initiation.The energy sensors, mTOR and AMP-activated kinase (AMPK), are the primary regulators of autophagy. AMPK activation or mTOR inhibition induces autophagy. Activated AMPK phosphorylates ULK1/2, and autophagy is then initiated. Various autophagy modulators and their targets are shown in the figure.

The initiation stage of autophagy is controlled by a series of autophagy-related genes (ATGs) [36]. Specifically, AMPK, mTOR, and unc-51-like kinase 1 (ULK1, also known as Atg1) play a central role in autophagy initiation [37] (Figure 2). Under conditions of nutrient sufficiency, active mTOR inhibits AMPK activation and phosphorylates ULK1 at the S758 site in humans (Ser757 in mouse) [38]. Phosphorylation of ULK S758 was also found to inhibit the interaction between ULK1 and AMPK. Upon nutrient starvation conditions, activated AMPK phosphorylates ULK1 at various sites and initiates autophagy [39]. Additionally, autophagy is accelerated by AMPK-induced phosphorylation of Raptor and TSC1, which is associated with mTOR inhibition [40]. Firstly, an autophagy activator can regulate autophagy initiation and serve as a treatment for cancer. This is described in Figure 2 and Table 1.

### 3.1. AMPK Activator

Activating AMPK is believed to be a promising cancer therapeutic because AMPK is involved in regulating cell growth-related pathways such as cell proliferation, protein synthesis and lipid biosynthesis [41]. AMPK acts as a sensor of AMP/ATP or ADP/ATP ratio and contributes to maintaining homeostasis when cellular energy is low [42].

Metformin, which has already been used for a long time as a drug against type 2 diabetes, induces autophagy by activating AMPK indirectly. Inhibition of mitochondrial respiratory chain complex I by metformin increases the AMP/ATP ratio, which then induces AMPK activation [43]. Several studies have suggested that metformin suppresses cell proliferation and induces apoptosis in several cancer cells, including renal, colorectal, liver and pancreatic cancer cells [21,44,45,46]. An in vivo study demonstrated that administration of metformin to hamsters with high-fat diet has diminished the occurrence of pancreatic cancer following exposure to pancreatic carcinogens [47]. Additionally, patients with type 2 diabetes who were given metformin had a significantly lower risk of developing pancreatic and hepatocellular cancer than patients who received other medications [48]. These studies suggest that AMPK activation and subsequent autophagy induction may have a preventive role in cancer incidence. Based on these studies, metformin is currently undergoing various clinical trials for cancer patients without diabetes [49]. Interestingly, the addition of metformin to standard EGFR-TKIs therapy in patients with advanced lung adenocarcinoma significantly improved progression-free survival in phase 2 randomized clinical trials [50]. Additionally, various phase 1 clinical trials were conducted for patients with glioblastoma or advanced/refractory cancer, and the stability of metformin was confirmed [51,52].

5-Aminoimidazole-4-carboxamide-1-β-d-ribofuranoside (AICAR), another activator of AMPK, induces apoptosis of renal cancer cells; moreover, AICAR is also known to inhibit autophagy by a pathway independent of AMPK [53]. Similar to other AMPK activators such as metformin, phenformin, salicylate and 2-deoxy-D-glucose (2-DG), AICAR inhibits cell growth; however, it also inhibits cell growth in AMPK-deficient cells, suggesting that AMPK-independent regulation exists [54,55]. In other words, AICAR may have nonspecific effects on AMPK or control independent of autophagy. Recently developed AMPK activators are more specific. A-769662, an AMPK activator, showed specific AMPK-dependent retardation of cell growth and metabolism [54]. GSK621, a direct and specific activator of AMPK, induces cytotoxicity and autophagy in acute myeloid leukemia (AML) [56]. Continuous efforts are made to develop specific and potent AMPK activators and use them for cancer treatment [57].

### 3.2. mTOR Inhibitor

Rapamycin/sirolimus, a representative mTOR inhibitor and autophagy inducer, was initially approved as an immunosuppressant to prevent allograft rejection. Due to its poor solubility and pharmacokinetics of rapamycin, several rapamycin analogs (rapalogs) were developed. Autophagy is induced in cancer cells not only by rapamycin but also by rapalogs [58]. Temsirolimus, a rapalog, was approved by the Food and Drug Administration (FDA) in 2007 for treating advanced renal cancer carcinoma (RCC). Thereafter, everolimus was approved for RCC treatment by FDA in 2009. Additionally, everolimus was later approved as a treatment for various cancers, including astrocytoma, breast cancer, angiomyolipoma and neuroendocrine cancer. However, rapalogs have achieved only modest effects in clinical practice. The reasons for clinical limit have not been fully established, but it may involve multiple mTORC1 regulatory negative feedback loops in cancer cells. To overcome this limitation, multiple clinical trials are currently evaluating the efficacy of rapalogs plus chemotherapy combination therapy [59]. AZD-8055 is a potent bioavailable ATP-competitive mTORC1 and mTORC2 inhibitor and is under phase I clinical trial in patients with various tumors. In preclinical data, AZD8055 exhibited a more potent anticancer effect than rapamycin in the brain tumor xenograft model [60]. Efforts focused on developing novel mTOR inhibitors or optimal combinations with rapalogs will have great potential to yield an improved efficacy.

Nutraceutical products obtained from normal food can control autophagy. Epigallocatechin gallate (EGCG), a polyphenol compound obtained from green tea, has been reported to modulate autophagy by affecting the balance of the mTOR-AMPK pathway [61,62,63]. Fisetin, a member of the flavonoid group of polyphenols, inhibits mTOR activity and induces autophagic-programmed cell death in several cancer cells, including prostate carcinoma and oral squamous cell carcinoma [64,65].

Various inhibitors of autophagy initiation (autophagy blockers) were developed and actively used in biological experiments to determine the role of autophagy in cancer cells. However, autophagy blockers have not entered clinical trials yet. Since various preclinical experiments are in progress, it is expected that autophagy blockers that enter clinical trials will emerge when numerous data are accumulated. Autophagy blockers are discussed below in terms of cancer treatment (Figure 2 and Table 1).

### 3.3. ULK1/2 Inhibitors

Several ULK1 or 2 activators, such as BL-918, typically target Parkinson’s disease but not cancers. Rather, ULK1/2 inhibitors have been developed as oncolytic drugs in certain cancers. In preclinical data, SBI-0206965, ULK1 inhibitor, reduced cell growth and promoted apoptosis in neuroblastoma [66]. SBI-0206965 and MRT-68921, another ULK1 inhibitor, was able to induce apoptosis in AML with FLT3-ITD mutation [67]. However, ULK1 inhibitors alone are not sufficient for tumor suppression in certain cancers. Recently, a paper was published explaining the reason why the anticancer effect of ULK inhibition monotherapy was ineffective in pancreatic ductal adenocarcinoma (PDAC) [68]. Intriguingly, autophagy inhibition induced micropinocytosis, which is the uptake of extracellular fluid droplets containing proteins and other macromolecules. As micropinocytosis provides energy to autophagy-compromised cells, ULK inhibition alone could not induce apoptosis in PDAC. Indeed, combination treatment of MRT-68921 and macropinosome formation inhibitor synergistically induced tumor regression in an in vivo model.

### 3.4. PI3K Inhibitors

Although the PI3K-AKT-mTOR axis is also critical for autophagy regulation, modulators of upstream kinases are not specific in autophagy regulation and show mixed autophagy readouts depending on cell types or treatment concentration and time. For example, 3-methyladenine (3-MA) is used as an autophagy inhibitor, but it also inhibits PI3K class I and class III. PI3K class III stimulates autophagic sequestering, whereas PI3K class I inhibits it [5]. Although the overall effect of 3-MA is considered an autophagy inhibitor, PI3K inhibitors are not suitable for clinical setting due to the complexity of the results.

## 4. Modulators of Lysosomal Activity

**Figure 3:**
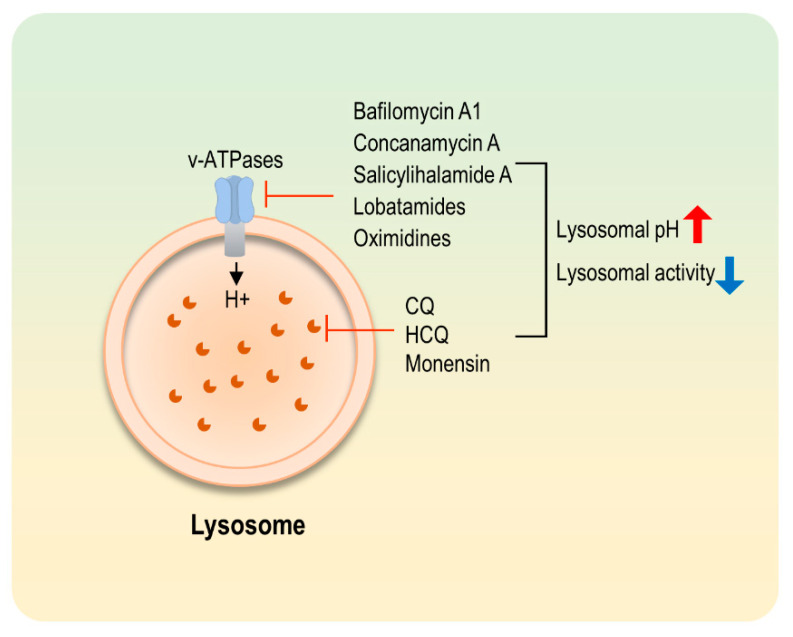
Lysosomal inhibitors. Autophagy is inhibited by lysosomal inhibitors. Lysosomal activity is inhibited by increasing intralysosomal pH or inhibiting v-ATPase.

Autophagosome fuses with a lysosome and eventually degrades the contents, including cargo proteins or organelles. Chloroquine (CQ) and a derivative of CQ, that is, hydroxychloroquine (HCQ), are the most well-known and clinically used chemical blockers (Figure 3). CQ and HCQ have been used as antimalarial drugs. CQ and HCQ inhibit autophagy by increasing intralysosomal pH, but the exact mechanism is not well understood. Autophagy inhibition limits the energy supply for cancer growth. Additionally, several damaged proteins or mitochondria can be accumulated in CQ- or HCQ-treated cells, resulting in the ER stress. Triggering excessive ER stress is an excellent strategy to kill cancer cells [69]. Apoptosis is eventually induced because cancer cells cannot tolerate excessive ER stress. In this context, CQ and HCQ show synergistic enhancement when combined with various therapeutic agents in cancer treatment. Treatment with CQ or HCQ along with radiation therapy or standard therapy is already in clinical trials. There are multiple reports that CQ sensitizes glioblastoma to radiation or chemotherapy. Favorable toxicity of CQ in patients was determined [70], and phase III clinical trials are ongoing (NCT00224978) [71]. According to the results of a recently published phase II clinical trial, HCQ along with gemcitabine and nab-paclitaxel resulted in a greater pathological tumor response in pancreatic cancer [72] (Table 2).

Vacuolar-type proton adenosine triphosphatase (v-ATPase) is an ATP driven proton pump responsible for controlling the acidification of lysosomes. Bafilomycin A1 and concanamycin A are v-ATPase inhibitors known to prevent the acidification of lysosomes (Figure 3). Several in vivo xenograft mouse studies revealed that bafilomycin A1 can inhibit tumor growth, including bladder, breast and liver cancers [73,74,75]. Although bafilomycin A1 is more famous and commonly used in many preclinical experiments to identify an inhibitory role in cancer growth, several more selective and potent v ATPase inhibitors exist (e.g., salicylihalamide A, lobatamides and oximidines). Unlike bafilomycin A1 and concanamycin A, these chemicals inhibit mammalian V-ATPases at low concentrations but do not affect fungi and yeast v-ATPases [76,77]. However, there are few reports that these chemicals inhibit tumor growth and autophagy, but further studies are still needed to confirm these findings (Table 2).

## 5. Modulation of CMA in Cancer

The recognition of substrates initiates CMA with KFERQ-like motifs by cytosolic chaperone Hsc70, and the substrates are then delivered to the lysosomal surface. The targeted substrates bind to lysosomal-associated membrane protein 2A (LAMP2A) and are translocated inwardly across the lysosomal membrane and are eventually degraded [4] (Figure 1). The role of CMA in oncology has not been fully understood as encompassing both pro-survival and pro-death parts in different contexts [78].

Similar to macroautophagy, CMA plays a critical supportive role in cancer cell proliferation because it satisfies the excessive energy needs of cancer cells by degradation of substrates. However, given CMA substrates, CMA degrades not only oncogenic proteins (e.g., PKM2, mutant p53, HK2, HIF-1α) but also tumor suppressors (unphosphorylated PED, Rnd3) [79]. These observations suggest that the role of CMA in oncology is more controversial than macroautophagy, especially regarding cancer treatment.

The distinct and notable characteristics of CMA are the selectivity of the target substrate and specificity of core components. CMA degrades only substrates with KFERQ-like motifs. Oncoproteins or oncoprotein activators with the KFERQ motif might be a promising therapeutic target through CMA modulation [45]. Hsc70 and LAMP2A are identified as the core components required for CMA. Specifically, LAMP2A is the rate-limiting component of the CMA pathway [80]. LAMP2A is under the negative regulation by nuclear retinoic acid receptor-α (RAR-α) [81]. Indeed, RAR-α inhibition by synthetic derivatives of all-trans-retinoic acid activated only CMA without regulating other RAR-α-dependent transcriptions [81]. However, RAR-α derivatives have the disadvantage that they can be used only when cancer cells are under the regulation of RAR-α. The application of RAR-α derivatives to cancer cells has not been published. Development of more diverse CMA modulators is required for application to anticancer treatment.

## 6. Autophagy Modulators Associated with Epigenetics

Although autophagy is rapidly regulated and occurs in the cytoplasm by starvation signal, transcriptional and epigenetic regulation in the nucleus are also deemed essential for the overall autophagy process [82]. Nutrient starvation regulates not only upstream kinase of autophagy, such as AMPK or mTOR, but also the translocation of transcription factors to the nucleus or histone modifications of target genes. Transcriptional regulation of autophagy is required to maintain prolonged autophagy. Furthermore, because autophagy receptors and LC-3 proteins are degraded with cargo, cells replenish autophagy proteins with transcriptional upregulation of autophagy-related genes.

### 6.1. MiT/TFE Family

The major transcription factor of autophagy regulation is the transcription factor EB (TFEB) [83]. TFEB belongs to the microphthalmia/transcription factor E (MiT/TFE) family of transcription factors that bind coordinated lysosomal expression and regulation element [84]. Under starvation conditions, TFEB translocates to the nucleus to promote the transcription of lysosome biogenesis and autophagy-related genes. Its phosphorylation status at various sites tightly controls the nuclear import or export of TFEB and induced by mTORC1 or GSK3β [85,86]. According to an interesting publication, increased mRNA and protein expression of MITF, TFE3 and TFEB was detected in pancreatic ducal adenocarcinoma (PDA) cell lines and patient tumors [87]. PDA is one of the most lethal of cancers with a 5-year survival rate of only 6% after diagnosis [88]; it is a cancer determined to be highly dependent on autophagy for the supply of essential nutrients. Overexpressed MiT/TFE family transcription factor activates autophagy and maintains intracellular amino acid pools. However, small molecules that can directly modulate the MiT/TFE family have not yet been developed. Since the MiT/TFE family is an attractive target for PDA treatment, it is expected to be an excellent therapeutic agent if potent and selective modulators are developed.

### 6.2. Histone Modifications

**Figure 4:**
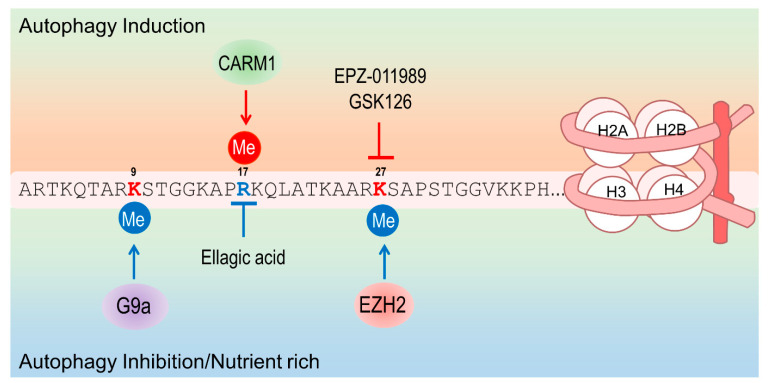
Histone H3 modifications associated with autophagy regulation.Histone H3 tail is methylated by G9a, EZH2 or CARM1. The epigenetic modulators can induce autophagy (inhibitor of H3K27 me) or block induced autophagy (inhibitor of H3R17 me).

Autophagy regulates, directly or indirectly, histone modifications to control transcription. Negative regulation occurs through histone H3 K9 or K27 methylation (Figure 4 and Table 3). Under nutrient-rich conditions, H3K9 methylation is maintained by G9a methyltransferase in autophagy-related genes [89]. However, a decrease in G9a recruitment and subsequent H3K9 dimethylation is observed by starvation. EZH2, a methyltransferase of H3 K27, regulated the expression of mTOR pathway-related genes through H3K27 methylation and knockdown of EZH2-induced autophagy [90]. In addition, EZH2 inhibitors such as GSK126 and EPZ-011989 induced autophagy [90,91] (Table 3). Interestingly, the epigenetic inhibitor induces autophagy by altering the transcription of the target genes. Moreover, in vivo studies revealed that these EZH2 inhibitors showed significant tumor growth inhibition in lymphoma and myeloma [92,93]. However, studies on how autophagy induction by EZH2 inhibitors affected overall tumor growth inhibition remain lacking.

Histone H3R17 methylation is associated with autophagy induction (Figure 4). Under nutrient starvation conditions, stabilized CARM1 methylated H3R17 and activated autophagy and lysosomal genes [94]. As CARM1 methyltransferase activity on H3R17 is inhibited by ellagic acid [95], which is a naturally occurring phenolic acid abundant in fruits and vegetables, treatment with ellagic acid was able to block autophagy induction even in starvation conditions (Table 3). Furthermore, ellagic acid inhibited pancreatic cancer growth in the xenograft model [96].

There are many reports that histone deacetylase (HDAC) inhibitors regulate autophagy [97], but the effects are a little complex and controversial (Table 3). It has been reported that SAHA/vorinostat, one of the HDAC inhibitors, inhibits mTOR and induces autophagy [97]. Additionally, treatment with SAHA improved autophagy flux by enhancing α-tubulin acetylation and alleviated Cockayne syndrome (CS), a condition of premature aging mainly caused by mutations in the *csb* gene [98]. Currently, SAHA is approved to treat T-cell lymphoma (CTCL), malignant mesothelioma and multiple myeloma. In the case of cancer in which reduced autophagy promotes tumorigenesis, treatment with SAHA can be effective. Another HDAC inhibitor, that is, MGCD0103/mocetinostat, has been reported to inhibit autophagy [99]. MGCD0103 decreased the autophagic flux in primary lymphocytic leukemia cells by activating the mTOR pathway and downregulating autophagy gene transcription [99]. This suggests that since the role of autophagy is different for each cancer type, it is crucial to identify the detailed function of autophagy according to cancer type and select an appropriate therapeutic agent. In addition, epigenetic regulators can control not only histone modification but also non-histone modification. If HDAC inhibitors modulate histone acetylation, overall transcription may be activated, but it remains difficult to predict how transcription will alter if they modulate an unknown non-histone target. Since target specificity can vary depending on the type of drug as well as the concentration and treatment time of the drug, a fundamental study on how and which target is controlled before application to cancer patients is required.

## 7. Conclusions and Future Perspective

Clever cancer cells use autophagy in a direction favorable to cancer survival, adaptation and rapid proliferation. Therefore, targeting autophagy is no doubt an excellent strategy for cancer therapy. However, the controversial role of autophagy should be kept in mind. Autophagy activation can be an excellent strategy in treating early stages of cancer. Toxic mutagen or genetic defects are eliminated by autophagy to maintain cellular homeostasis. However, autophagy can also support the growth of cancer cells as tumors develop. Additionally, cancer cells use autophagy to meet their increased metabolic demands by recycling macromolecules and supplying building blocks. Accordingly, autophagy modulators should be applied to consider various cancer characteristics such as cancer development stage, cancer types, cancer microenvironments and oncogenic mutations. Of course, it is necessary to determine the state of autophagy of cancer before applying activators or inhibitors.

Many clinical trials using autophagy modulators are combinatorial therapy with standard chemotherapy or radiotherapy. Since enhanced or impaired autophagy in cancer is one of the various characteristics of cancers, it will be difficult to show dramatic anticancer effects with autophagy modulator alone. However, the effect of disappearing chemotherapy resistance caused by prolonged standard treatments is positive by inducing an imbalance of autophagy. These effects may be due to the induction of apoptosis, alterations in the tumor microenvironment or a still unknown mechanism. More detailed mechanistic studies of how autophagy modulators overcome chemotherapy resistance are needed for the stronger efficacy of autophagy modulators.

It is interesting that epigenetic drugs such as EZH2 inhibitors or HDAC inhibitors regulate autophagy. However, the specific mechanism by which epigenetic drugs affect autophagy remains largely unknown. Since the effect of autophagy in cancer depends on cancer type and tumor stage, it is challenging to determine whether autophagy regulation by epigenetic drugs will have a positive/negative effect on cancer treatment. Therefore, it is necessary to study the autophagy mechanisms and treatment outcomes of epigenetic drugs in a wider range of cancers.

Metformin has been used for a long time in patients with type 2 diabetes and has been clinically proven to be safe. Moreover, HCQ and CQ have been used in patients with malaria. Repurposing drugs with potential antitumor properties might have the advantage of improving survival while saving time and money. These drugs have successfully passed phase I clinical trials for cancer patients and are currently undergoing phases II/III. It is expected for use in the treatment of cancer patients after its effectiveness and safety have been confirmed.

Recently, immunotherapy is attracting attention as a new cancer treatment. So far, the synergistic effect of autophagy and antibody-targeted therapy has frequently been reported [100]. However, autophagy also enhances or attenuates the effectiveness of immunotherapy, depending on the cancer type [93]. Currently, phase I/II clinical trials on HCQ in combination with nivolumab/ipilimumab in advanced melanoma are recruiting patients (NCT04464759). If positive clinical results are obtained, it is expected that the effective range of autophagy modulators as cancer immunotherapeutics will be further expanded.

## Figures and Tables

**Table 1:** Autophagy modulators associated with autophagy initiation.

Compound	Mechanism/Use	Structure	Clinical Trials in Progress or Completed for Cancer Treatment (NCT Number)
Metformin	AMPK activation/autophagy inducer	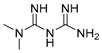	Phase 3 in breast cancer (NCT01101438) Phase 3 in colorectal cancer (NCT02614339) Phase 3 in endometrial cancer (NCT02065687)
AICAR	AMPK activation, AMP analog/autophagy inducer	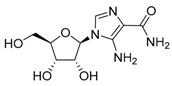	Preclinical
A-769662	AMPK activation/autophagy inducer	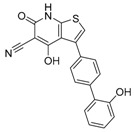	Preclinical
GSK621	AMPK activation/autophagy inducer	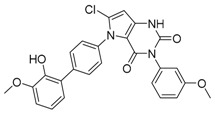	Preclinical
Rapamycin/sirolimus	mTOR inhibition/autophagy inducer	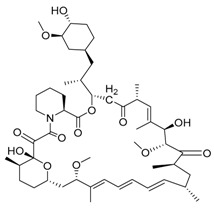	Approved to treat lymphangioleiomyomatosis (LAM)
Temsirolimus/CCI-779	mTOR inhibition, rapalog/autophagy inducer	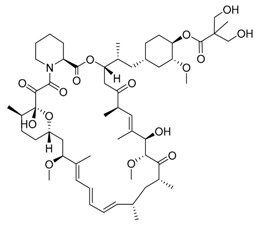	Approved for the treatment of renal cell carcinoma
Everolimus/RAD001	mTOR inhibition, rapalog/autophagy inducer	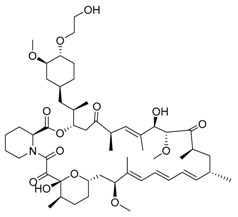	Approved for the treatment of renal cell carcinoma, advanced breast cancer, pancreatic cancer, renal angiomyolipoma, tuberous sclerosis complex, neuroendocrine tumors (NET), advanced gastrointestinal tumors
Ridaforolimus/MK-8669	mTOR inhibition, rapalog/autophagy inducer	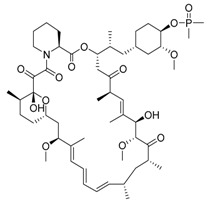	Withdrawn from the European Union market in 2012
AZD8055	mTORC1/2 inhibition/autophagy inducer	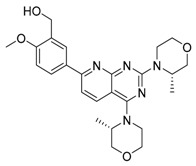	Phase 1 in recurrent gliomas (NCT01316809) Phase 1 in liver cancer (NCT00999882) Phase 1 in advanced solid tumors (NCT00731263, NCT00973076)
Torin 1	mTORC1/2 inhibition/autophagy inducer	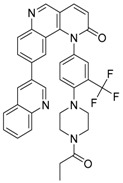	Preclinical
MRT-68921	ULK1/2 inhibition/autophagy inhibitor	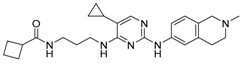	Preclinical
SBI-0206965	ULK1 inhibition/autophagy inhibitor	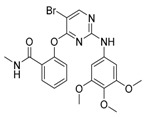	Preclinical
3-Methyladenine (3-MA)	Class I and III PI3K inhibition/autophagy inhibitor, but autophagy effects depend on concentration or cell type	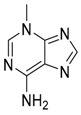	Preclinical
Wortmannin	Non-specific, covalent inhibition of PI3K/autophagy inhibitor	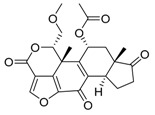	Preclinical
EGCG	Modulating balance between mTOR and AMPK/autophagy inducer or inhibitor (different roles depending on concentration)	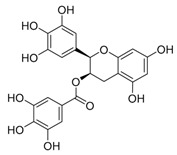	Nutraceutical
Fisetin	mTOR inhibition/autophagy inducer	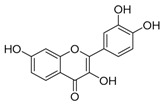	Nutraceutical

**Table 2:** Autophagy modulators that control lysosomal activity.

Compound	Mechanism/Use	Structure	Clinical Trials in Progress or Completed for Cancer Treatment (NCT Number)
CQ	Inhibition of lysosomal acidification/autophagy inhibitor	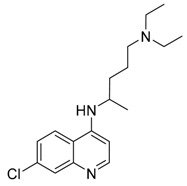	Phase 3 in glioblastoma (NCT00224978)/ Phase 1/2 in IDH1/2-mutated solid tumors (NCT02496741) Phase 1/2 in ductal carcinoma in situ (DCIS) (NCT01023477) Phase 2 in breast cancer (NCT01446016)
HCQ	Inhibition of lysosomal acidification/autophagy inhibitor	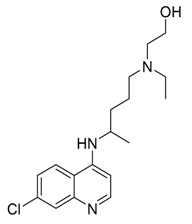	Phase 2 in Pancreatic cancer (NCT01273805, NCT01494155, NCT01978184, NCT01128296) Phase 2 in colorectal cancer (NCT01006369, NCT01206530 NCT02316340, NCT03215264) Phase 2 in lung cancer (NCT00977470, NCT01649947, NCT02470468) Phase 2 in renal cell carcinoma (NCT01510119, NCT01550367)
Bafilomycin A1	Inhibition of v-ATPase/autophagy inhibitor	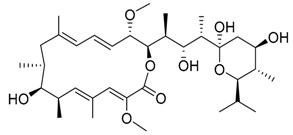	Preclinical
Concanamycin A	Inhibition of v-ATPase/autophagy inhibitor	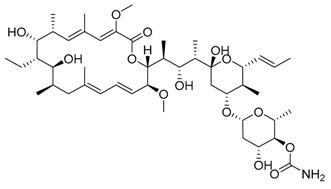	Preclinical
Salicylihalamide A	Inhibition of v-ATPase/no autophagy research	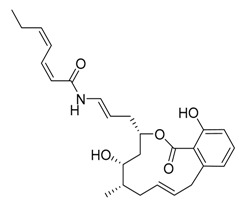	No autophagy research

**Table 3:** Autophagy modulator associated with epigenetics.

Compound	Mechanism/Use	Structure	Clinical Trials in Progress or Completed for Cancer Treatment (NCT Number)
GSK126	EZH2 inhibition/autophagy inducer	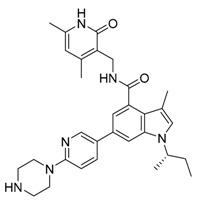	Discontinued in phase 1 (solid tumors and lymphoma)
EPZ-011989	EZH2 inhibition/autophagy inducer	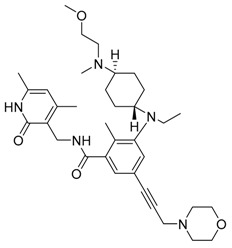	Preclinical
Ellagic acid	CARM1 inhibition/blocking autophagy induction	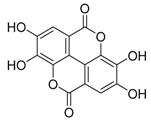	Preclinical
SAHA/vorinostat	HDAC inhibition/autophagy inducer	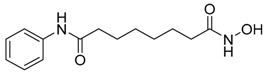	Approved for the treatment of T-cell lymphoma (CTCL)
MGCD0103/mocetinostat	HDAC inhibition/autophagy inhibitor	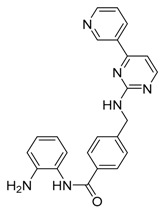	Phase 2 in lung cancer (NCT02954991) Phase 2 in urothelial carcinoma (NCT02236195) Phase 2 in lymphoma (NCT02282358, NCT02429375, NCT00359086)

## Data Availability

Not applicable.
